# Interleukin-7 regulates CD127 expression and promotes CD8^+^ T cell activity in patients with primary cutaneous melanoma

**DOI:** 10.1186/s12865-022-00509-0

**Published:** 2022-07-18

**Authors:** Hongxia He, Binjun Qiao, Shuping Guo, Hongzhou Cui, Ziyan Zhang, Junxia Qin

**Affiliations:** 1 Department of Dermatology, The First Hospital of Shanxi Medical University, 85 South Jiefang Road, Taiyuan, 030000 Shanxi China; 2 Department of Emergency, The First Hospital of Shanxi Medical University, Taiyuan, 030000 Shanxi China; 3 Department of Dermatology, The Affiliated Shanxi Provincial People’s Hospital of Shanxi Medical University, Taiyuan, 030000 Shanxi China

**Keywords:** CD127, CD8^+^ T lymphocytes, Immune regulation, Interleukin-7, Melanoma

## Abstract

**Background:**

Interleukin (IL)-7 signaling through CD127 is impaired in lymphocytes in cancers and chronic infections, resulting in CD8^+^ T cell exhaustion. The mechanisms underlying CD8^+^ T cell responses to IL-7 in melanoma remain not completely elucidated. We previously showed reduced IL-7 level in melanoma patients. Thus, the aim of this study was to investigate the effect of IL-7 regulation on CD127 expression and CD8^+^ T cell responses in melanoma.

**Methods:**

Healthy controls and primary cutaneous melanoma patients were enrolled. Membrane-bound CD127 (mCD127) expression on CD8^+^ T cells was determined by flow cytometry. Soluble CD127 (sCD127) protein level was measured by ELISA. Total CD127 and sCD127 mRNA level was measured by real-time PCR. CD8^+^ T cells were stimulated with recombinant human IL-7, along with signaling pathway inhibitors. CD8^+^ T cells were co-cultured with melanoma cell line, and the cytotoxicity of CD8^+^ T cells was assessed by measurement of lactate dehydrogenase expression.

**Results:**

Plasma sCD127 was lower in melanoma patients compared with controls. The percentage of CD8^+^ T cells expressing mCD127 was higher, while sCD127 mRNA level was lower in peripheral and tumor-infiltrating CD8^+^ T cells from melanoma patients. There was no significant difference of total CD127 mRNA expression in CD8^+^ T cells between groups. IL-7 stimulation enhanced total CD127 and sCD127 mRNA expression and sCD127 release by CD8^+^ T cells. However, mCD127 mRNA expression on CD8^+^ T cells was not affected. This process was mainly mediated by phosphatidylinositol 3-kinase (PI3K) pathway. CD8^+^ T cells from melanoma patients exhibited decreased cytotoxicity. IL-7 stimulation promoted CD8^+^ T cell cytotoxicity, while inhibition of PI3K dampened IL-7-induced elevation of CD8^+^ T cell cytotoxicity.

**Conclusion:**

The current data suggested that insufficient IL-7 secretion might contribute to CD8^+^ T cell exhaustion and CD127 dysregulation in patients with primary cutaneous melanoma.

## Background

There are more than 0.3 million newly diagnosed patients melanoma of skin, which accounts for 0.6% of cancer-related death in 2020 all over the world [1]. The incidence of primary cutaneous melanoma, which is one of the most aggressive malignancies in humans, continues to increase and is responsible for 65% of skin cancer deaths due to the high metastatic ability [2, 3]. The early diagnosis and appropriate therapy for cutaneous melanoma leads to a cure rate of more than 90%. However, advanced stages of cutaneous melanoma always results in poor outcome, which made it a pivotal area of research for development of new therapeutics [4]. Importantly, immune checkpoint inhibitors administration achieves essential clinical benefits in malignant melanoma [5, 6].

Interleukin (IL)-7 is a member of common γ chain receptor cytokine family, and is important for naïve T cell differentiation and survival, as well as memory T cell development and homeostasis [7–9]. Our previous study has been demonstrated that plasma IL-7 was lower in melanoma patients, which was insufficient for maintenance of Th17 cell activation [10]. IL-7 receptor is a heterodimer, and is composed of the common γ chain receptor and IL-7-specific α chain (CD127) [11, 12]. The degree of CD127 expression determines the extent of signal through IL-7/IL-7 receptor complex, which activates two major pathways: Janus kinase/signal transducer and activator of transcription 5 (JAK/STAT5) and Akt/phosphatidylinositol 3-kinase (Akt/PI3K) [13, 14]. Furthermore, IL-7 is known to modulate CD127 expression. There are two forms of CD127 existed, including membrane-bound CD127 (mCD127) on immune cells and soluble CD127 (sCD127) in peripheral blood. Exogenous IL-7 stimulation reduces total CD127 and mCD127 expression in T cells in vitro [15–17] and in vivo [18]. IL-7 also mediates the release of sCD127 from T cells through two potential methods [16]. On the one hand, IL-7 induced an alternative mRNA splicing pathway for CD127. The splicing process leads to the removal of exon 6 encoding the transmembrane domain for CD127, and generates the truncated protein sCD127 [19, 20]. On the other hand, mCD127 could be digested and shed from membrane of immune cells to generate sCD127 mainly by specific matrix metalloproteinases (MMP) [17, 20]. During human immunodeficiency virus (HIV)-1 infection, IL-7 mediates sCD127 release and mCD127 down-regulation in human CD8^+^ T cells [20]. This process is impaired and results in CD8^+^ T cell dysfunction [20]. Due to the impairment of CD8^+^ T cell responsiveness to IL-7 in chronic infections [20, 21] and cancers [22], we sought to test the hypothesis that altered responsiveness to IL-7 in circulating and tumor-infiltrating CD8^+^ T cells and CD127 expression is one of the probable mechanisms for immune exhaustion in patients with primary cutaneous melanoma.

## Methods

### Patients and controls

This study was approved in compliance with the Declaration of Helsinki by the Ethics Committee at The First Hospital of Shanxi Medical University (Approval number: 2016-KY-08072 and 2021-K-K102). Written informed consents were obtained from all enrolled patients and controls. The sample size numbers were calculated by Clinical Research Sample Size Calculator. Thirty-eight patients with primary cutaneous melanoma (median age, 43 years; range, 27–68 years; 27 men and 11 women; stage, stage I: 19, stage II: 11; stage III: 8) were enrolled from The First Hospital of Shanxi Medical University between July 2018 and December 2020. The diagnosis of melanoma was made in accordance with the clinical manifestation and was confirmed by pathological examination. All patients were treatment-naïve, who did not receive anti-tumor or immuno-modulatory therapies before sampling. The exclusion criteria were the following: (1) patients with chronic hepatitis virus and HIV-1 infection; (2) patients with autoimmune disorder; (3) patients with other malignancies; (4) patients with severe liver or renal dysfunction; (5) patients with pregnancy. Twenty-two age- and sex-matched healthy individuals (median age, 42 years; range, 22–63 years; 14 men and 8 women) were also enrolled as controls. All enrolled subjects were Chinese Han population.

### Isolation of peripheral blood mononuclear cells and tumor-infiltrating lymophocytes

Peripheral blood mononuclear cells (PBMCs) were isolated with Ficoll-Hypaque density centrifugation reagent Histopaque-1077 (Sigma-Aldrich, St Louis, Missouri, USA). Tumor tissues and para-tumor tissues were obtained from fourteen patients with primary cutaneous melanoma who received surgery. Tumor-infiltrating lymophocytes (TILs) were isolated as previously described [10]. Briefly, 1.3–2 g of tumor or para-tumor tissue was cut into small pieces and passage through 70-µm pore strainers. Cells were treated with collagenase D (500 µg/mL) at 37 °C in 5% CO_2_ condition for a 30 min digestion. The digested cells were then re-suspended in 44% Percoll (Sigma-Aldrich) in RPMI1640 (*vol*/*vol*), and were layed over 56% Percoll in PBS (*vol*/*vol*). The gradient was centrifuged at 850×*g* for 30 min. The interphase containing TILs was collected and washed with RPMI1640. A total of (2.4–8.8) × 10^5^ of TILs were harvested from each tissue sample.

### Enrichment of CD8^+^ T cells

CD8^+^ T cells were purified from PBMCs or TILs by using human CD8^+^ T cell isolation kit (Miltenyi Biotec, Bergisch Gladbach, Germany). The purity post enrichment of CD8^+^ T cells was 95.7 ± 2.4%. Purified CD8^+^ T cells were cultured in DMEM supplemented with 10% fetal bovine serum, penicillin (100 µg/mL), L-glutamine (2 mmol/L), and anti-CD3/CD28 (1 µg/mL) at 37 °C in 5% CO_2_ condition.

### Cell stimulation and culture

Purified CD8^+^ T cells were stimulated with recombinant human IL-7 (10 ng/mL; R&D Systems, Minneapolis, Minnesota, USA) [22] for 48 h in the presence of anti-CD3/CD28, along with either JAK inhibitor (10 µmol/L; Sigma-Aldrich, Temecula, California, USA), STAT5 inhibitor (250 µmol/L; Merck Millipore), or PI3K inhibitor (LY294002) (25 µmol/L; Sigma-Aldrich) as previously described [20]. Control cells were only stimulated with anti-CD3/CD28 for maintenance of CD8^+^ T cell survival. In certain experiments, stimulated CD8^+^ T cells were washed twice. 10^4^ of CD8^+^ T cells were co-cultured with 10^5^ of melanoma cell line SK-MEL-5 cells for 48 h.

### Enzyme-linked immunosorbent assay

sCD127 level in the plasma and supernatant was measured by Human Soluble Interleukin-7 Receptor, sIL-7R enzyme-linked immunosorbent assay (ELISA) kit (CUSABIO, Wuhan, Hubei Province, China). Interferon-γ (IFN-γ) and tumor necrosis factor-α (TNF-α) level in the supernatants was measured by Human IFN-γ ELISA kit (CUSABIO) and Human TNF-α ELISA kit (CUSABIO), respectively.

### Flow cytometry

PBMCs or TILs were stained with anti-human CD8-APC (Clone 3B5; Invitrogen ThermoFisher, Carlsbad, California, USA), and anti-human CD127-PE-Cyanine5 (Clone eBioRDR5; eBioscience, Invitrogen ThermoFisher, San Diego, California, USA) at room temperature for 30 min in the dark. Cells were detected by a FACS Aria II flow cytometer (BD Bioscience, San Jose, California, USA), and analyzed by FlowJo V10 software (FlowJo LCC, TreeStar, Ashland, Oregon, USA).

### Real-time PCR

Total RNA was extracted from CD8^+^ T cells using Trizol reagent (Invitrogen ThermoFisher) according to the instructions from the manufacturer. cDNA was synthesized with random hexamers using PrimeScript RT Master Mix (TaKaRa, Beijing, China). For total CD127 mRNA (including RNA encoding both mCD127 and sCD127) quantification, the primers covered exons 5–7 was designed previously [20]. The PCR was able to amplify full-length CD127 RNA that skipped exon 6. This PCR amplification was performed using TB Green *Premix Ex Taq* II (Tli RNaseH Plus) (TaKaRa). The relative total CD127 mRNA expression to Glyceraldehyde-3-phosphate dehydrpgenase was measured using 2^*−*ΔΔ*CT*^ method with ABI7500 System Sequence Detection software (Applied Biosystems, Foster, CA, USA). For sCD127 mRNA variant detection, the probe covered nucleotides 777–815 of the variant (lack of exon 6) was previously designed [20]. The PCR amplification was performed using *Premix Ex Taq* (Probe qPCR) (TaKaRa), and analyzed using ABI7500 System Sequence Detection software (Applied Biosystems).

### Cytotoxicity analysis

The cytotoxicity of CD8^+^ T cells to target cells were calculated by measurement of lactate dehydrogenase (LDH) expression in the supernatants by using LDH cytotoxicity assay kit (Beyotime, Wuhan, Hubei Province, China). LDH level in the supernatant of SK-MEL-5 cells was defined as “low-level control”, while LDH level in the supernatant of Triton X-100 treated SK-MEL-5 cells was defined as “high-level control”. The percentage of target cells = (LDH level in the sample − low-level control)/(high-level control − low-level control) × 100%.

### Statistical analysis

Statistical analysis was performed by using SPSS 21.0 (SPSS, Chicago, Illinois, USA). Homogeneity test of variances was firstly performed, and all parameters were followed with normal distribution, which were presented as mean ± standard deviation. Statistical differences between the two groups were determined by Student’s *t* test. Statistical differences between multi-groups were determined by one-way ANOVA. LSD-*t* test was used after one-way ANOVA. All tests were two-tailed, and *P* < 0.05 was considered to indicate a statistically significant difference.

## Results

### mCD127 expression on CD8^+^ T cells was higher, while sCD127 was lower in patients with primary cutaneous melanoma

**Fig. 1:**
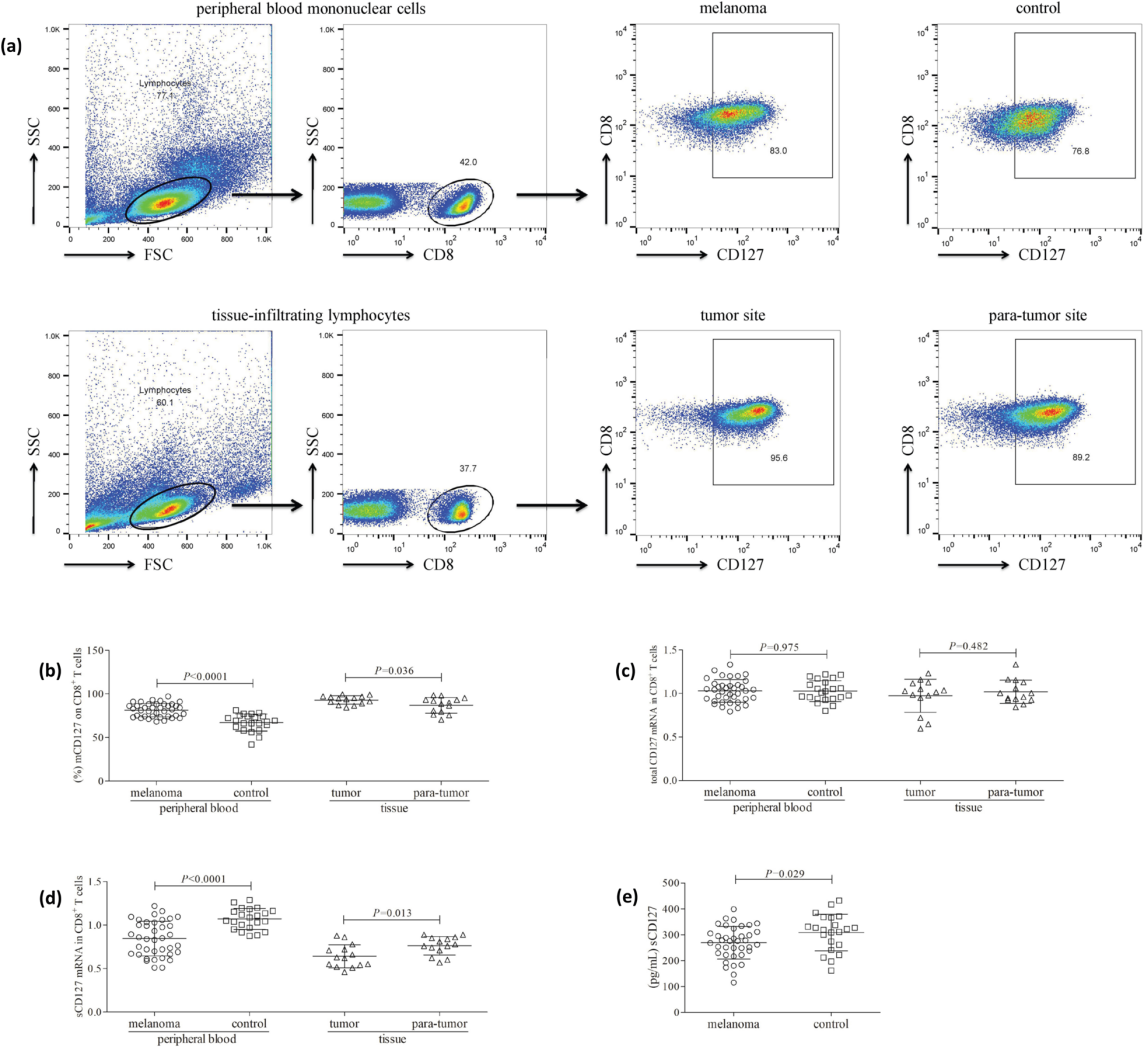
Membrane-bound CD127 (mCD127) on CD8^+^ T cells was higher, while soluble (sCD127) was lower in patients with primary cutaneous melanoma. Peripheral blood mononuclear cells were isolated from all enrolled subjects (38 patients with primary cutaneous melanoma and 22 healthy controls), while tissue-infiltrating lymphocytes were isolated from tumor and para-tumor tissues of 14 melanoma patients with surgical operation. Cells were stained with anti-CD8 and anti-CD127, and were analyzed by flow cytometry. **a** The representative flow cytometry analyses for peripheral blood mononuclear cells and tissue-infiltrating lymphocytes are shown. Live lymphocytes were gated according to forward scatter (FSC) and side scatter (SSC). mCD127 expression within CD8^+^ T cells were assessed. **b** The percentage of peripheral CD8^+^ T cells expressing mCD127 was elevated in melanoma patients compared with controls. The percentage of tissue-infiltrating CD8^+^ T cells expressing mCD127 was also increased in tumor tissues compared with para-tumor tissues. CD8^+^ T cells were purified from peripheral bloods and tissue-infiltrating lymphocytes. Total CD127 mRNA and sCD127 mRNA variant in CD8^+^ T cells was measured by real-time PCR. **c** Total CD127 mRNA level in peripheral CD8^+^ T cells was comparable between melanoma patients and controls. Total CD127 mRNA level in tissue-infiltrating CD8^+^ T cells was also comparable between tumor tissues and para-tumor tissues. **d** sCD127 mRNA level in peripheral CD8^+^ T cells was lower in melanoma patients compared with controls. sCD127 mRNA level in tissue-infiltrating CD8^+^ T cells was also lower in tumor tissues compared with para-tumor tissues. **e** sCD127 expression in the plasma was measured by ELISA. Plasma sCD127 level was lower in melanoma patients compared with controls. Individual level of each subject is shown. Statistical analysis was performed using Student’s *t* test

Live lymphocytes were gated according to forward scatter and side scatter. mCD127 expression on CD8^+^ T cells was analyzed in both PBMCs and TILs (Fig. 1a). The percentage of peripheral CD8^+^ T cells expressing mCD127 was elevated in patients with primary cutaneous melanoma (81.32 ± 7.68%) when compared with healthy controls (67.05 ± 9.72%; *P* < 0.0001, Fig. 1b). mCD127 expression on CD8^+^ T cells in tumor and para-tumor tissue was investigated in fourteen patients with primary cutaneous melanoma. The proportion of tissue-infiltrating CD8^+^ T cells expressing mCD127 was also increased in tumor tissues (92.94 ± 4.86%) when compared with para-tumor tissues (86.89 ± 9.00%; *P* = 0.036, Fig. 1b). There was no significant difference of total CD127 mRNA either in peripheral CD8^+^ T cells between melanoma patients and controls (1.03 ± 0.13 vs. 1.03 ± 0.12; *P* = 0.975, Fig. 1c), or in tissue-infiltrating CD8^+^ T cells between tumor tissues and para-tumor tissues (0.97 ± 0.19 vs. 1.02 ± 0.14; *P* = 0.482, Fig. 1c). sCD127 mRNA level in peripheral CD8^+^ T cells was lower in melanoma patients (0.85 ± 0.20) when compared with controls (1.07 ± 0.12; *P* < 0.0001, Fig. 1d). sCD127 mRNA level in tissue-infiltrating CD8^+^ T cells was also reduced in tumor tissues (0.64 ± 0.13) when compared with para-tumor tissues (0.76 ± 0.11; *P* = 0.013, Fig. 1d). Plasma sCD127 expression was lower in melanoma patients (269.2 ± 63.24 pg/mL) when compared with controls (308.7 ± 70.58 pg/mL; *P* = 0.029, Fig. 1e).

### IL-7 stimulation induced sCD127 release without affecting mCD127 expression on CD8^+^ T cells in patients with primary cutaneous melanoma and controls

**Fig. 2:**
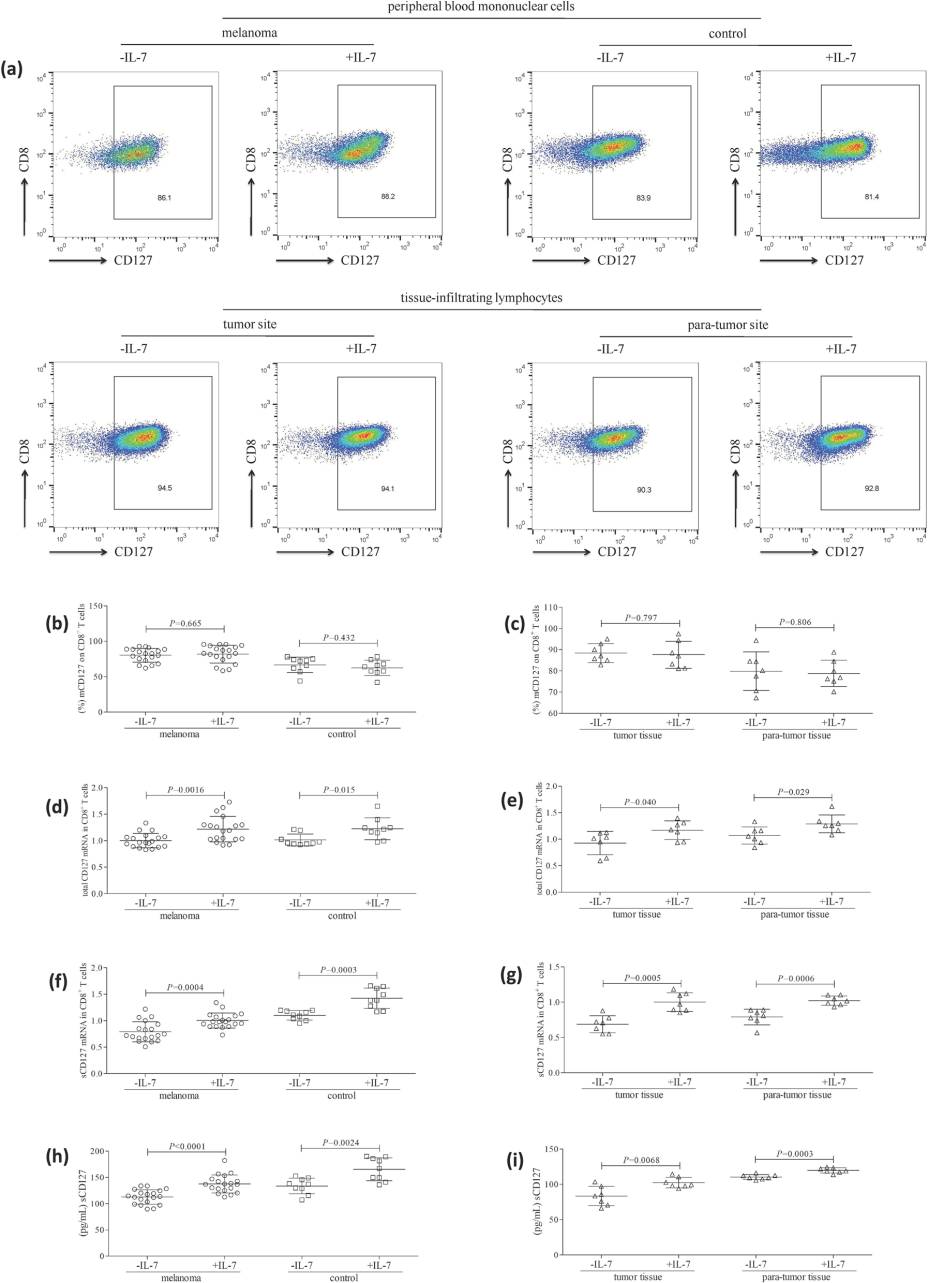
Recombinant human IL-7 stimulation did not affect mCD127 expression on CD8^+^ T cells, but induced sCD127 release in patients with primary cutaneous melanoma and controls. 10^4^ of peripheral CD8^+^ T cells (nineteen patients with primary cutaneous melanoma and nine healthy controls) and tissue-infiltrating CD8^+^ T cells (tumor and para-tumor tissues of seven melanoma patients) were stimulated with recombinant human IL-7 (10 ng/mL) for 48 h in the presence of anti-CD3/CD28. Control cells were cultured with anti-CD3/CD28 only. Cells and supernatants were harvested. **a** mCD127 expression on CD8^+^ T cells was investigated by flow cytometry, and the representative flow dots are shown. **b** There was no significant difference of the percentage of peripheral CD8^+^ T cells expressing mCD127 between cells with and without IL-7 stimulation in either melanoma patients or controls. **c** There was no remarkable difference of the percentage of tissue-infiltrating CD8^+^ T cells expressing mCD127 between cells with and without IL-7 stimulation in either tumor or para-tumor tissues. **d** IL-7 stimulation enhanced total CD127 mRNA level in peripheral CD8^+^ T cells in both melanoma patients and controls. **e** IL-7 stimulation enhanced total CD127 mRNA level in tissue-infiltrating CD8^+^ T cells in both tumor tissues and para-tumor tissues. **f** IL-7 stimulation promoted sCD127 mRNA level in peripheral CD8^+^ T cells in both melanoma patients and controls. **g** IL-7 stimulation promoted sCD127 mRNA level in tissue-infiltrating CD8^+^ T cells in both tumor tissues and para-tumor tissues. **h** IL-7 stimulation promoted sCD127 secretion by peripheral CD8^+^ T cells in both melanoma patients and controls. **i** IL-7 stimulation promoted sCD127 secretion by tissue-infiltrating CD8^+^ T cells in both tumor tissues and para-tumor tissues. Individual level of each subject is shown. Statistical analysis was performed using Student’s *t* test

10^4^ of purified CD8^+^ T cells from patient with primary cutaneous melanoma and controls were stimulated with recombinant human IL-7 (10 ng/mL) for 48 h in the presence of anti-CD3/CD28. Control cells were cultured with anti-CD3/CD28 only. The representative flow dots for mCD127 expression in peripheral and tissue-infiltrating CD8^+^ T cells are shown in Fig. 2a. There was no significant difference of the percentage of peripheral CD8^+^ T cells expressing mCD127 between cells with and without IL-7 stimulation in either melanoma patients or controls (*P* > 0.05, Fig. 2b). Similarly, IL-7 stimulation did not affect the percentage of tissue-infiltrating CD8^+^ T cells expressing mCD127 in either tumor or para-tumor tissues (*P* > 0.05, Fig. 2c). IL-7 stimulation enhanced total CD127 mRNA level both in peripheral CD8^+^ T cells from melanoma patients (1.22 ± 0.24 vs. 1.00 ± 0.14; *P* = 0.0016, Fig. 2d) and controls (1.23 ± 0.21 vs. 1.01 ± 0.11; *P* = 0.015, Fig. 2d), as well as in tissue-infiltrating CD8^+^ T cells from tumor tissues (1.17 ± 0.18 vs. 0.93 ± 0.22; *P* = 0.040, Fig. 2e) and para-tumor tissues (1.29 ± 0.17 vs. 1.07 ± 0.16; *P* = 0.029, Fig. 2e). Similarly, IL-7 stimulation also promoted sCD127 mRNA level both in peripheral CD8^+^ T cells from melanoma patients (1.00 ± 0.14 vs. 0.79 ± 0.19; *P* = 0.0004, Fig. 2f) and controls (1.42 ± 0.19 vs. 1.10 ± 0.09; *P* = 0.0003, Fig. 2f), as well as in tissue-infiltrating CD8^+^ T cells from tumor tissues (1.00 ± 0.13 vs. 0.69 ± 0.12; *P* = 0.0005, Fig. 2g) and para-tumor tissues (1.02 ± 0.07 vs. 0.79 ± 0.11; *P* = 0.0006, Fig. 2g). IL-7 mediated the elevation of sCD127 secretion by peripheral CD8^+^ T cells from both melanoma patients (137.6 ± 17.09 pg/mL vs. 112.9 ± 13.84 pg/mL; *P* < 0.0001, Fig. 2h) and controls (165.7 ± 21.91 pg/mL vs. 133.8 ± 15.10 pg/mL; *P* = 0.0024, Fig. 2h). IL-7 also induced sCD127 secretion by tissue-infiltrating CD8^+^ T cells from both tumor tissues (83.44 ± 13.59 pg/mL vs. 102.5 ± 7.47 pg/mL; *P* = 0.0068, Fig. 2i) and para-tumor tissues (119.9 ± 3.67 pg/mL vs. 110/5 ± 3.45 pg/mL; *P* = 0.0003, Fig. 2i).

### IL-7-induced sCD127 release by CD8^+^ T cells could be inhibited by PI3K blockade

**Fig. 3:**
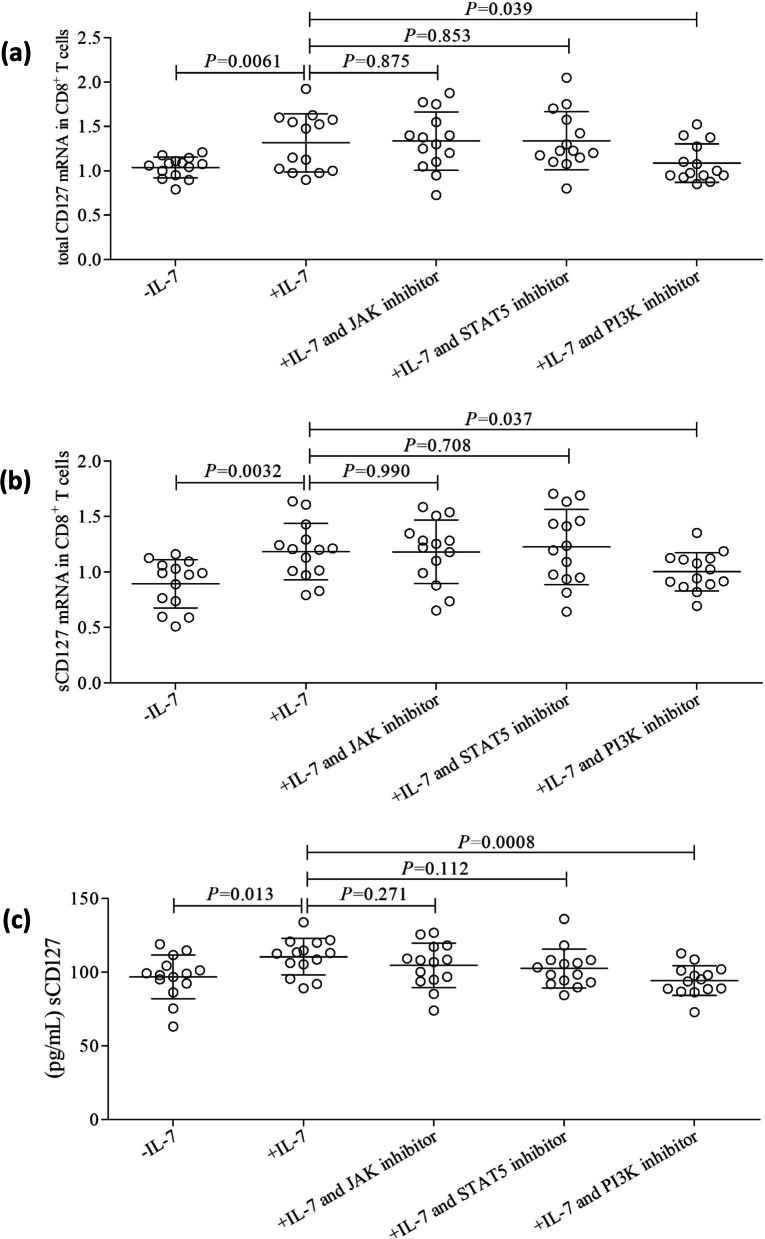
Influence of JAK, STAT5, and PI3K inhibitor to IL-7-mediated CD127 expression in CD8^+^ T cells in patients with primary cutaneous melanoma. 10^4^ of purified peripheral CD8^+^ T cells from fourteen patient with primary cutaneous melanoma were stimulated with recombinant human IL-7 (10 ng/mL) for 48 h in the presence of anti-CD3/CD28, along with either JAK inhibitor (10 µmol/L), STAT5 inhibitor (250 µmol/L), or PI3K inhibitor (25 µmol/L). Control cells were cultured with anti-CD3/CD28 only. Cells and supernatants were harvested. Total CD127 mRNA and sCD127 mRNA level in CD8^+^ T cells were measured by real-time PCR. **a** IL-7 stimulation promoted total CD127 mRNA expression in CD8^+^ T cells. Either JAK inhibitor or STAT5 inhibitor did not affect IL-7-mediated elevation of total CD127 mRNA expression, while PI3K inhibitor abrogated the effect of IL-7 on total CD127 mRNA induction in CD8^+^ T cells. **b** IL-7 stimulation also enhanced sCD127 mRNA level in CD8^+^ T cells. Either JAK inhibitor or STAT5 inhibitor did not influence IL-7-induced elevation of sCD127 mRNA expression, while PI3K inhibitor abrogated the effect of IL-7 on sCD127 mRNA induction in CD8^+^ T cells. **c** sCD127 level in the supernatants was measured by ELISA. IL-7 stimulation promoted sCD127 level in the cultured supernatant of CD8^+^ T cells. Either JAK inhibitor or STAT5 inhibitor did not influence IL-7-induced sCD127 secretion, while PI3K inhibitor abrogated the effect of IL-7 on sCD127 production by CD8^+^ T cells. Individual level of each subject is shown. Statistical analysis was performed using one-way ANOVA and LSD-*t* test

JAK, STAT5, and PI3K are primary activated signaling pathways following the binding of IL-7 to IL-7 receptor complex [13, 14]. To investigate the mechanisms by which IL-7 modulates the CD127 secretion by CD8^+^ T cells, 10^4^ of purified peripheral CD8^+^ T cells from fourteen patient with primary cutaneous melanoma were stimulated with recombinant human IL-7 (10 ng/mL) for 48 h in the presence of anti-CD3/CD28, along with either JAK inhibitor (10 µmol/L), STAT5 inhibitor (250 µmol/L), or PI3K inhibitor (25 µmol/L). Control cells were cultured with anti-CD3/CD28 only. Inhibition of either JAK or STAT5 did not affect IL-7-mediated elevation of total CD127 mRNA expression (Fig. 3a), sCD127 mRNA level (Fig. 3b), or sCD127 production by CD8^+^ T cells (Fig. 3c) (*P* > 0.05). Importantly, PI3K inhibitor abrogated the effect of IL-7 on total CD127 mRNA (Fig. 3a), sCD127 mRNA expression (Fig. 3b), or sCD127 secretion by CD8^+^ T cells (Fig. 3c) (*P* < 0.05).

### IL-7-induced enhancement of CD8^+^ T cell cytotoxicity could be suppressed by PI3K blockade

**Fig. 4:**
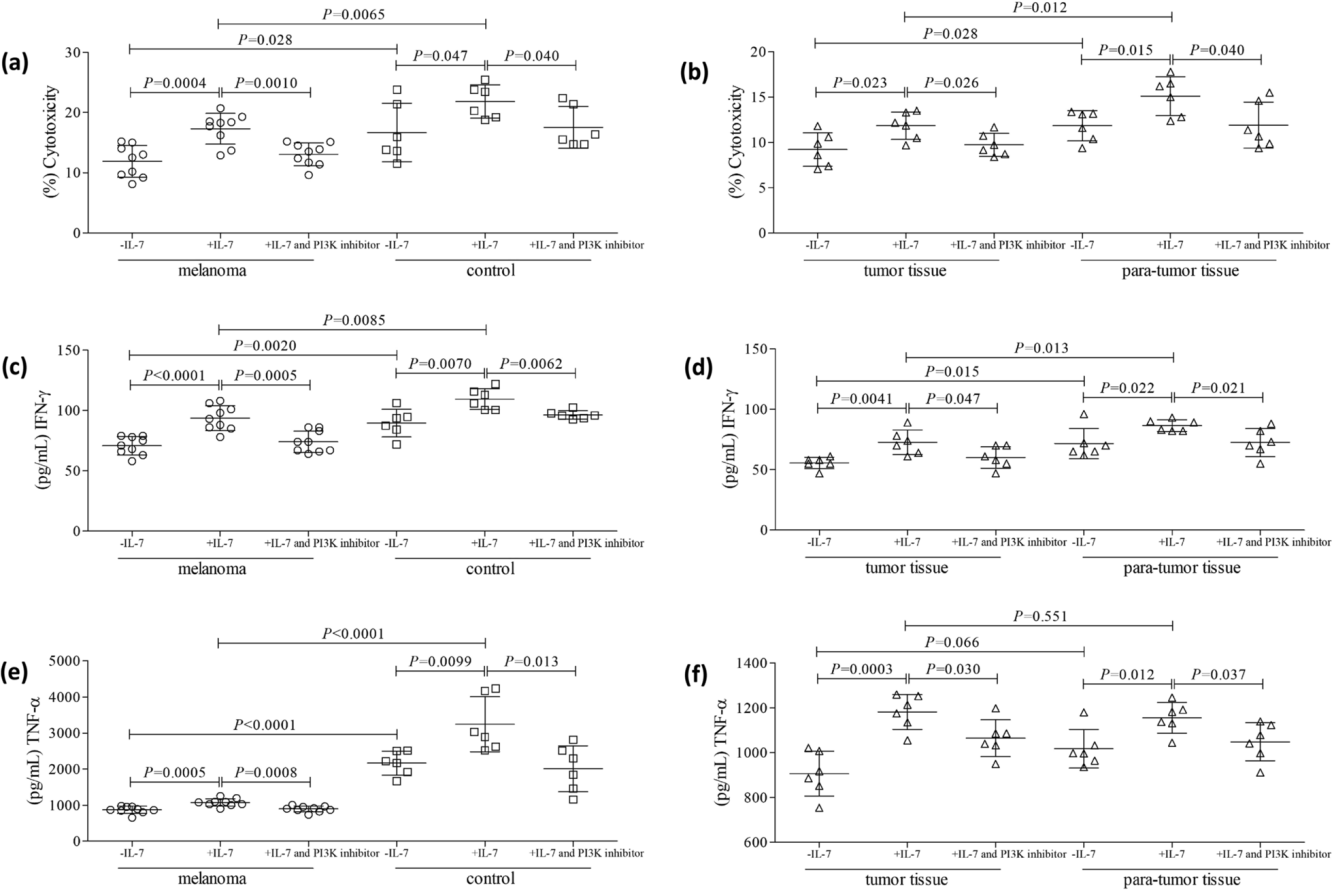
Influence of PI3K inhibitor to IL-7-mediated CD8^+^ T cell cytotoxicity in patients with primary cutaneous melanoma. Purified peripheral CD8^+^ T cells (nine HLA-A02 restricted patient with primary cutaneous melanoma and six HLA-A02 restricted healthy individuals) and tissue-infiltrating CD8^+^ T cells (tumor and para-tumor tissues of six HLA-A02 restricted melanoma patients) were stimulated with recombinant human IL-7 (10 ng/mL) and PI3K inhibitor (25 µmol/L) for 48 h in the presence of anti-CD3/CD28. Cells were washed twice, and 10^4^ of stimulated CD8^+^ T cells were co-cultured with 10^5^ of SK-MEL-5 cells for another 48 h. The percentage of target cell death was calculated by measurement of LDH level in the supernatants. IFN-γ and TNF-α level in the supernatants was measured by ELISA. Inhibition of PI3K dampened the cytotoxicity of **a** peripheral CD8^+^ T cells from melanoma patients and controls, as well as **b** tissue-infiltrating CD8^+^ T cells from tumor tissues and para-tumor tissues. Inhibition of PI3K inhibitor suppressed IFN-γ secretion by **c** peripheral CD8^+^ T cells from melanoma patients and controls, as well as **d** tissue-infiltrating CD8^+^ T cells from tumor tissues and para-tumor tissues. Inhibition of PI3K inhibitor also suppressed TNF-α secretion by **e** peripheral CD8^+^ T cells from melanoma patients and controls, as well as **f** tissue-infiltrating CD8^+^ T cells from tumor tissues and para-tumor tissues. Individual level of each subject is shown. Statistical analysis was performed using one-way ANOVA and LSD-*t* test

Purified CD8^+^ T cells from HLA-A02 restricted patient with primary cutaneous melanoma and HLA-A02 restricted healthy individuals were stimulated with recombinant human IL-7 (10 ng/mL) and PI3K inhibitor (25 µmol/L) for 48 h in the presence of anti-CD3/CD28. Cells were washed twice, and 10^4^ of stimulated CD8^+^ T cells were co-cultured with 10^5^ of SK-MEL-5 cells for 48 h. Peripheral CD8^+^ T cells presented decreased cytotoxicity from melanoma patients, which mediated reduced target cell death compared with healthy individuals (11.88 ± 2.64% vs. 16.67 ± 4.86%; *P* = 0.028, Fig. 4a). Similarly, tissue-infiltrating CD8^+^ T cells also showed reduced cytotoxicity from tumor tissues compared with para-tumor tissues (11.85 ± 1.67% vs. 9.23 ± 1.85%; *P* = 0.028, Fig. 4b). IL-7 promoted the cytotoxicity of peripheral CD8^+^ T cells from melanoma patients (*P* = 0.0004, Fig. 4a) and controls (*P* = 0.047, Fig. 4a), as well as tissue-infiltrating CD8^+^ T cells from tumor tissues (*P* = 0.023, Fig. 4b) and para-tumor tissues (*P* = 0.015, Fig. 4b). The cytotxocity of CD8^+^ T cells was still lower in peripheral blood and tumor tissues in melanoma patients (*P* < 0.05, Fig. 4a, b). PI3K inhibitor abrogated IL-7-induced elevation of peripheral and tissue-infiltrating CD8^+^ T cells in vitro (*P* < 0.05, Fig. 4a, b). IFN-γ and TNF-α level in the supernatants was measured by ELISA. IL-7 enhanced both IFN-γ (*P* < 0.05, Fig. 4c, d) and TNF-α (*P* < 0.05, Fig. 4e,f) expression in the cultured supernatants of peripheral and tissue-infiltrating CD8^+^ T cells co-culture system. PI3K inhibitor also abrogated IL-7-induced IFN-γ (*P* < 0.05, Fig. 4c, d) and TNF-α (*P* < 0.05, Fig. 4e, f) secretion by peripheral and tissue-infiltrating CD8^+^ T cells.

## Discussion

In the present study, we firstly screened the expression profile of sCD127 and mCD127 expression in CD8^+^ T cells in patients with primary cutaneous melanoma. Circulating sCD127 was lower, while mCD127 expression on CD8^+^ T cells was higher in melanoma patients. Meanwhile, sCD127 mRNA variant was also lower in melanoma patients, although there was no significant difference of total CD127 mRNA variant between melanoma patients and healthy individuals. Moreover, IL-7 is known to regulate CD127 expression in CD8^+^ T cells through multiple mechanisms in vitro [17, 19, 20], and we previously reported the decreased expression of peripheral IL-7 in melanoma patients [10]. Thus, we further investigated the modulatory activity of exogenous IL-7 to CD127 expression and cytotoxicity of circulating and tissue-infiltrating CD8^+^ T cells in melanoma patients. We found that recombinant human IL-7 stimulation promoted sCD127 release by CD8^+^ T cells through PI3K signaling pathway. This process was accompanied by the elevation of both sCD127 mRNA and total CD127 mRNA in CD8^+^ T cells, but mCD127 expression on CD8^+^ T cells was not affected. Inhibition of PI3K abrogated IL-7-induced enhancement of CD8^+^ T cell cytotoxicity in both melanoma patients and healthy individuals. The current data revealed that IL-7 contributed to cytotoxicity of CD8^+^ T cells and regulation of CD127 expression.

The expression of IL-7 receptor complex on the immune cells not only determines the responsiveness of the cells to IL-7, but also showed the consuming efficiency of cell to IL-7 [23]. Thus, chronic infection and cancers might induce the changes in CD127 expression profile, leading to the immune tolerance or evasion for viral persistence or tumor metastasis. However, CD127 expression profile was divergently reported in various diseases. Chronic hepatitis C patients had lower mCD127 level on circulating CD8^+^ T cells [21]. Similarly, reduced mCD127 expression on T cells was closely associated with decreased CD4^+^ T cell counts and increased viral replication in HIV-1-infected patients [24]. There was no significant difference of mCD127 expression on peripheral or liver-infiltrating CD8^+^ T cells between hepatocellular carcinoma patients and controls [22]. However, number of T cells expressing CD127 was reduced in peripheral blood of patients with breast cancer, resulting in IL-7 signaling defects [25]. Herein, we showed that there was imbalance between sCD127 and mCD127 expression on CD8^+^ T cells in melanoma patients. In contrast to previous findings, our results revealed lower level of circulating sCD127 and higher expression of mCD127 on CD8^+^ T cells in melanoma patients. Meanwhile, sCD127 mRNA, but not total RNA, was also reduced in CD8^+^ T cells in melanoma patients, indicating that melanoma might mainly suppress sCD127 release from CD8^+^ T cells, resulting in the elevation of mCD127 expression on CD8^+^ T cells without impacting total CD127 level. However, the role of alternative expression profile of CD127 in melanoma is still need further elucidated.

The mechanisms involved in the regulation of sCD127/mCD127 expression are not fully understood. In vitro IL-4 stimulation suppressed IL-7-mediated STAT5 phosphorylation, leading to reduction in mCD127 expression and mature CD8^+^ T cells proliferation [26]. sCD127 secretion by purified CD8^+^ T cells was also dependent on MMP-9 activity, which did not affect mCD127 expression on the cell surface in HIV-infected patients [20]. IL-7 could mediate alternative CD127 expression profile in various diseases. In this study, the regulatory function of IL-7 to CD127 expression in melanoma patients was investigated. In the previous study on IL-7 regulation to CD8^+^ T cells in HCC patients, 10 ng/ml of IL-7 was used in vitro stimulation [22]. Similarly, Hou et al. used 5 ng/ml of IL-7 for CD8^+^ T cells stimulation in vitro in chronic hepatitis C virus-infected patients [21]. The higher concentration of exogenous IL-7 might be sufficient for cellular stimulation in cancers. Thus, we chose 10 ng/ml of recombinant IL-7 for CD8^+^ T cells stimulation in melanoma patients. Our present study revealed that exogenous IL-7 promoted the release of sCD127 by CD8^+^ T cells, but mCD127 expression on CD8^+^ T cells was not affected. This was controversial with the previous reports showing that in vitro human T cells expressed reduced mCD127 in the presence of exogenous IL-7 [16, 17, 20] and in vivo IL-7 administration led to the reduction of mCD127 expression on T cells [18, 27]. Most the above studies were performed in HIV-infected individuals. However, the regulatory role of IL-7 might be in a context-specific manner, and might be different in cancers. Thus, our current data suggested that IL-7-induced sCD127 release by CD8^+^ T cells might be not related to proteolytic cleavage and shedding of the membrane receptor as described with other cytokine receptors [28, 29], which was not consistent with the in vitro and in vivo regulatory mechanism between mCD100 and sCD100 balance in lung cancer [30] and chronic viral infections [31, 32]. Moreover, IL-7 also induced the pathway favoring the alternatively spliced version of CD127 gene, leading to the direct secretion of truncated sCD127. Meanwhile, total CD127 RNA level in CD8^+^ T cells was also increased in response to IL-7 stimulation. This indicated that IL-7 could also mediated sCD127 production via increased gene expression, resulting in transcriptional regulation of sCD127 and total CD127 gene. Previous studies also demonstrated post-transcriptional and translational modulation to mCD127 in mice [33]. Thus, it was likely that IL-7 might induce further pathways following translation of spliced sCD127 mRNA variant to elevate sCD127 release by CD8^+^ T cells. Furthermore, IL-7-induced sCD127 release by CD8^+^ T cells was mainly dependent on PI3K activity, but was independent of JAK/STAT5 signaling pathway. This was not consistent with our previous finding that in vitro regulation of Th17 response to IL-7 was STAT5 dependent in melanoma patients [10]. IL-7 receptor complex could activate PI3K/Akt/mTOR signaling pathway in lung cancer cell line and leukemia transformation cell line [34, 35]. IL-7 receptor-dependent PI3K also competed with STAT5 signal to regulate T cell development and homeostasis in mice [36]. Taken together, IL-7 induced an increase in sCD27 mRNA variant in CD8^+^ T cells and release of sCD127 by CD8^+^ T cells probably through PI3K signaling pathway.

CD8^+^ T cell exhaustion was one of the hallmarks in the immunopathogenesis of chronic viral infection and malignancies [37]. Our present data revealed the decreased cytotoxicity of both peripheral and tumor-infiltrating CD8^+^ T cells to target melanoma cell line in vitro, confirming the CD8^+^ T cell dysfunction or exhaustion in melanoma patients. Consistent with the previous finding in viral infection and cancers [22, 38–40], we found that IL-7 promoted peripheral and tissue-infiltrating CD8^+^ T cell activity in melanoma patients. CD8^+^ T cells exhibited cytotoxicity via two independent pathways. CD8^+^ T cells not only revealed direct cytolytic activity to target cells through perforin/granzyme secretion and Fas/FasL interaction, but also secreted pro-inflammatory cytokines, including IFN-γ and TNF-α, inducing cytokine-mediated cell necrosis or apoptosis [41, 42]. IL-7 stimulation to CD8^+^ T cells from melanoma patients not only enhanced direct cytolytic function of CD8^+^ T cells to target cells, but also promoted IFN-γ and TNF-α secretion, indicating that IL-7 increased cytolytic and non-cytolytic activity of CD8^+^ T cells in melanoma patients. Importantly, CD8^+^ T cell-induced cytotoxicity or IFN-γ production in response to IL-7 stimulation was still lower in peripheral blood and tumor tissue of melanoma patients. This might be due to the decreased responsiveness of CD8^+^ T cells to IL-7 in cancers, As previous findings showed that IL-7 did not affect tumor patients derived CD8^+^ T cell proliferation in vitro [22]. Furthermore, administration of PI3K inhibitor dampen IL-7-induced enhancement of CD8^+^ T cell cytotoxicity, which was closely related to CD127 regulation. TNF-α secretion by IL-7 stimulation was increased by in CD8^+^ cells from tumor and para-tumor tissue. However, PI3K inhibitor administration did not reduce the TNF-α level to untreated level. On the one hand, CD8^+^ T cell-induced target cells was significantly decreased to untreated level with IL-7 stimulation in combination with PI3K blockade, indicating that TNF-α might not be a key mediator for cytotoxicity of CD8^+^ T cells. On the other hand, IL-7 treatment increased α_4_β_7_ integrin expression on naïve human T cells [43, 44], resulting in the amplification of inflammation induced by other cytokines, primarily TNF-α [45]. IL-7 signaling pathway influence anti-TNF responsiveness and T cell gut homing in inflammatory bowel disease [43]. Transcription factor FoxO1, which ameliorated TNF-α-induced tissue damage [46], also served as the downstream of IL-7 signaling pathway and contributed to adenosine-mediated CD8^+^ T cells immunosuppression [47]. Thus, there might be another signaling pathway, which was activated by IL-7, leading to TNF-α production. Moreover, PI3K plays multiple activities both physiologically and pathologically. Thus, PI3K might impact sCD127 expression, while separately having an impact on the cytotoxic potential of CD8^+^ T cells. Taken together, the current data indicated that IL-7 promoted CD127 expression and the cytotoxic potential of CD8^+^ T cell in melanoma patients. However, the major limitation of the present work was the small sample size, which could be further addressed by in vivo experiments.

## Conclusion

In summary, insufficient IL-7 secretion might contribute to CD8^+^ T cell exhaustion and CD127 dysregulation in patients with primary cutaneous melanoma. IL-7 signaling through CD127 and PI3K pathway might be one of the potential therapeutic approaches to promote immune response for treatment of melanoma.

## Data Availability

The datasets generated and analyzed during the current study are not publicly available due to limitations of ethical approval involving the patient data and anonymity but are available from the corresponding author on reasonable request.

## Abbreviations

ELISA: Enzyme-linked immunosorbent assay
HIV: Human immunodeficiency virus
IFN-γ: Interferon-γ
IL: Interleukin
JAK/STAT5: Janus kinase/signal transducer and activator of transcription 5
LDH: Lactate dehydrogenase
mCD127: Membrane-bound CD127
MMP: Matrix metalloproteinases
PBMCs: Peripheral blood mononuclear cells
PI3K: Phosphatidylinositol 3-kinase
sCD127: Soluble CD127
TILs: Tumor-infiltrating lymophocytes
TNF-α: Tumor necrosis factor-α
